# Spatiotemporal control of DNA-based chemical reaction network *via* electrochemical activation in microfluidics

**DOI:** 10.1038/s41598-018-24659-7

**Published:** 2018-04-23

**Authors:** Ievgen Kurylo, Guillaume Gines, Yannick Rondelez, Yannick Coffinier, Alexis Vlandas

**Affiliations:** 1 BioMEMS, Univ. Lille, CNRS, ISEN, UMR 8520 - IEMN, F-59000 Lille, France; 2 NanoBioInterfaces, Univ. Lille, CNRS, ISEN, UMR 8520 - IEMN, F-59000 Lille, France; 3 Laboratoire Gulliver, Ecole Supérieure de Physique et de Chimie Industrielles, PSL Research University, and CNRS, Paris, France

## Abstract

In recent years, DNA computing frameworks have been developed to create dynamical systems which can be used for information processing. These emerging synthetic biochemistry tools can be leveraged to gain a better understanding of fundamental biology but can also be implemented in biosensors and unconventional computing. Most of the efforts so far have focused on changing the topologies of DNA molecular networks or scaling them up. Several issues have thus received little attention and remain to be solved to turn them into real life technologies. In particular, the ability to easily interact in real-time with them is a key requirement. The previous attempts to achieve this aim have used microfluidic approaches, such as valves, which are cumbersome. We show that electrochemical triggering using DNA-grafted micro-fabricated gold electrodes can be used to give instructions to these molecular systems. We demonstrate how this approach can be used to release at specific times and locations DNA- based instructions. In particular, we trigger reaction-diffusion autocatalytic fronts in microfluidic channels. While limited by the stability of the Au-S bond, this easy to implement, versatile and scalable technique can be used in any biology laboratory to provide new ways to interact with any DNA-based computing framework.

## Introduction

Living organisms perform complex computation at the cellular level through networks of intertwined chemical reactions^1^. In recent years, several groups have attempted to develop frameworks of chemical reactions networks (CRNs) which can run *in vitro*. Indeed, it is widely anticipated that the ability to produce ever more complex CRNs will allow not only for a better understanding of key biological processes but also enable the design of novel biosensors, smart autonomous systems, unconventional computation etc^2^.

Among the favoured approaches to produce synthetic CRNs^3^, the ones relying on DNA seem to have taken the lead^4^. DNA possesses a unique ability for high density information encoding^5^. Combined with its complementary-based programmable assembly, it provides a versatile way to design reaction cascades and control their kinetics. Furthermore, the ever decreasing cost of synthesizing DNA strands makes this approach an attractive choice as any successful applications could realistically be used widely. Using DNA for computation can be traced back to the seminal paper by Adleman^6^ which illustrated the power of the massively parallel processing of information permitted by DNA by solving the Hamiltonian path problem. Since then several CRNs DNA approaches have been proposed^7,8^. One of the most recent DNA computing framework, Polymerase/Exonuclease/Nickase Dynamic Network Assembly toolbox (or PEN DNA toolbox), was developed by Rondelez *et al*.^9,10^ and relies on a combination of short (10–20 base pairs) DNA strands and enzymes to create dynamical system which can be used for information processing.

Initially performed in a well-mixed solution in a tube inside a real-time PCR instrument, the PEN toolbox was thereafter also used in microfluidic channels under microscope observation to monitor its spatiotemporal behaviour. For example, it was demonstrated that reaction-diffusion waves could propagate in microfluidics set-ups using DNA autocatalytic amplification^11^ or predator-prey CRN^12^. In the natural world, reaction-diffusion phenomena are at the heart of various processes such as morphogenesis in which chemical reactions propagate in space to produce concentration patterns (either stable or transient). Recently, these complex phenomena were also reproduced artificially with the synthesis and materialization of a French flag pattern^13^.

Several issues remain in our view to be solved to fully exploit these discoveries and turn them into real life technologies. Of particular interest is the ability to interact in real-time with these complex molecular systems which  is a key requirement for their complexification. As information is materialized through DNA strands in these systems, the on-demand injection of DNA input strands might be a useful way to provide a versatile and controllable method to do so. There are several ways to make biomolecules available in a reaction medium that are compatible with microfluidics. A first approach is to inject in the main channel where information processing takes place a volume of solution which contains the input DNA strand. One can do so using microfluidic valves^14^ which can be opened and closed at will to enable the solutions to come in contact and exchange DNA strands through diffusion. This approach, however, requires multilayers microfluidics chips to be produced and is rather complex to operate. Furthermore, information exchange can only be done at a limited number of locations and always at the edges of a reservoir or channel. Rather than leaving the DNA input strands in solution, it is possible to use surface immobilization and release strategies. This can be done, for example, by photocleavage of photolabile linker molecules^15^, using an alternating electromagnetic field and Fe_3_O_4_ as a substrate^16^ or simple thermal desorption^17^. In the present work, our original approach proposes to use the electrochemical properties of the thiol-gold bond to release DNA from gold electrodes^18^. We prove that it can be easily combined with PEN toolbox system without changing the reaction conditions (temperature, ionic strength) and does not require expensive equipment. Furthermore, our method can be used to provide complex spatiotemporal actuation in microfluidics as one can pattern microelectrodes of various shapes and sizes in reservoirs or channels which can be addressed individually as required.

Using this strategy of on-demand electrical release, we have performed time- and space-controlled triggering of a DNA autocatalytic system based on PEN DNA toolbox, and initiated the propagation of reaction-diffusion fronts, as a way to demonstrate the possibility to couple electrical devices with a molecular information processing framework.

## Results and Discussion

### Reaction mechanism

**Figure 1:**
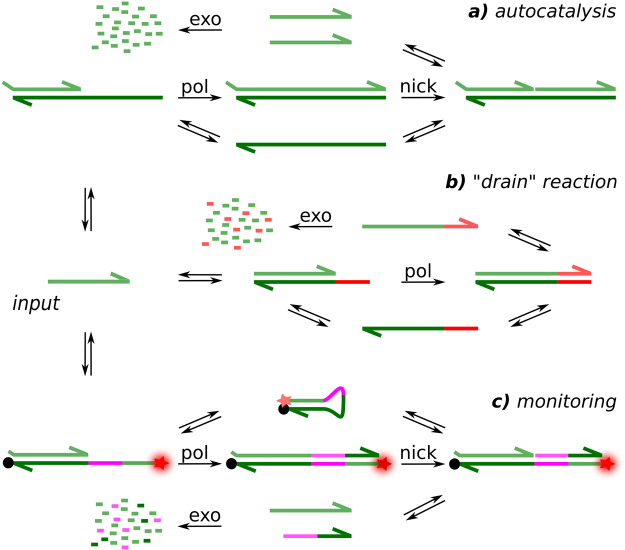
Schematic of the three reactions which constitutes the Chemical Reaction Network and its monitoring strategy. Subnetwork (**a**) is the autocatalysis core, (**b**) is the non-linear degradation pathway used to induce bistability and (**c**) the molecular beacon reporter whose intensity is proportional to the concentration of input strand.

Figure 1 shows the chemical reaction network (CRN) used in this study which is based on the PEN toolbox, a full description of which can be found here^19^. It is composed of sequences of DNA oligonucleotides, called templates, which encode the reactions and a mediator strand called input. It relies on three enzymes: a polymerase, a nickase and an exonuclease to produce an out-of-equilibrium CRN of two interconnected reactions. While the input and output strands can get degraded by the exonuclease, the templates are protected from this thanks to 3 phosphorothioate (PTO) modified nucleotides (nt) on their 5′ end.

The reaction, shown in Fig. 1a, is based on 22 nt DNA template which produces an autocatalytic amplification of the input. When an input strand hybridizes on the 3′ end, it gets elongated by the polymerase and cut by the nickase to produce 2 copies of itself. These in turn can be used to produce more copies of themselves. Such template thus induces an autocatalytic amplification reaction, which is a meta-stable in the sense that, even in the absence of initial trigger, it will always initiate spontaneously after some time^20^. A strategy is therefore required to prevent this self-start. To this end, the first input-binding part of the template is truncated by 2 nt to decrease its affinity to the input, and a second template is introduced.

This second template codes the reaction shown in Fig. 1b. In order to suppress untriggered start of the autocatalysis, an additional, non-linear, degradation pathway is added through this pseudo-template (pT). The pseudo-template is 17 nt long, with the same PTO modification as the template. It is fully complementary to the input on its 3′ end and thus presents a higher affinity to it than the autocatalytic template. After hybridization on the pT, the input strand is elongated by the polymerase. The last 5 nt are not complementary to the autocatalytic template and the elongated input strand is therefore no longer able to participate in autocatalytic reaction. Furthermore, the absence of the nickase recognition site does not allow for recycling of the initial input through cleavage. As a result, the elongated sequence can only be degraded by the exonuclease. This degradation pathway has a limited throughput, controlled by the concentration of the pseudo-template and its turnover rate. When this throughput is exceeded, it is not able to process the additional inputs, and the amplification process encoded by the autocatalytic template can proceed.

By tuning the template and pseudo-template concentrations ratio it is therefore possible to make autocatalytic reaction resistant for input concentrations lower than a chosen “threshold level” (Figs S5–7. The stabilisation of the low state, induced by the presence of the pseudo-template, is a key requirement to achieve a robust autocatalytic CRN which only starts when purposely triggered.

The last DNA sequence added to the mix enables monitoring in real time the input strand concentration. As shown in Fig. 1c, a sequence-specific DNA molecular beacon was used with a Cy-5 dye on its 5′ end and a BHQ2 quencher on its 3′ end. When the beacon is in its closed, self-hybridised, configuration, the proximity of the quencher to the dye prevents any fluorescence from being emitted. In presence of an input strand, the beacon is stabilized in its open configuration through the polymerase-mediated elongation of the input and fluorescence can be observed. This reporting strategy is reversible, because the nickase cuts the extended input strand part of the duplex – as it contains its recognition site – and recycles the molecular beacon and the input. Doing so it releases another inactive short strand which gets subsequently degraded. Consequently, the monitoring process, unlike the two reactions that compose the thresholded autocatalytic CRN, has little influence on the reaction kinetics (Fig. S8).

### Input release

In order to confirm our ability to successfully attach and release input at a designated time, a fluorescently-labelled DNA dithiol strand was used. While attached to the gold surface, the fluorophore is quenched and little fluorescence is observed. After the voltage pulse is performed, the DNA detaches from the surface; the fluorophore is no longer quenched and the corresponding fluorescence increase can be detected. Imaging the channel filled with solutions of the same fluorescently tagged DNA with known concentrations we can obtain a calibration table. Using it, we have determined the concentration on top of the working electrode after the electrical release to be equal to 1.80 ± 0.04 nM and, knowing the electrode surface area, calculated the surface coverage, using method similar to Demers *et al*.^21^. It amounted to ~10^10^ molecules/cm^2^ (Fig. S3), which is in line with previously reported values for low density DNA-thiol SAM on gold^22^.

While it can be tempting to maximize the surface density of the DNA grafting (Fig. S2), we find that higher densities are not actually desirable. In fact, thermal desorption of attached input can trigger the reaction even in the absence of an electrical pulse. Indeed, due to radical nature of the cleavage of Au-S bond mechanism^17^, the longer distance between the thiol groups, the less radical transfer occurs and the less likely desorption is. Fig. S11 shows that at 38 °C during 250 minutes no thermal desorption happened and the system remained stable. After 250 min slow detachment from the surface occurred. The desorption curve follows a linear trend from 250 min up to the end of monitoring time (1000 min).

### Triggering of autocatalytic CRN and initiation the reaction-diffusion fronts

**Figure 2:**
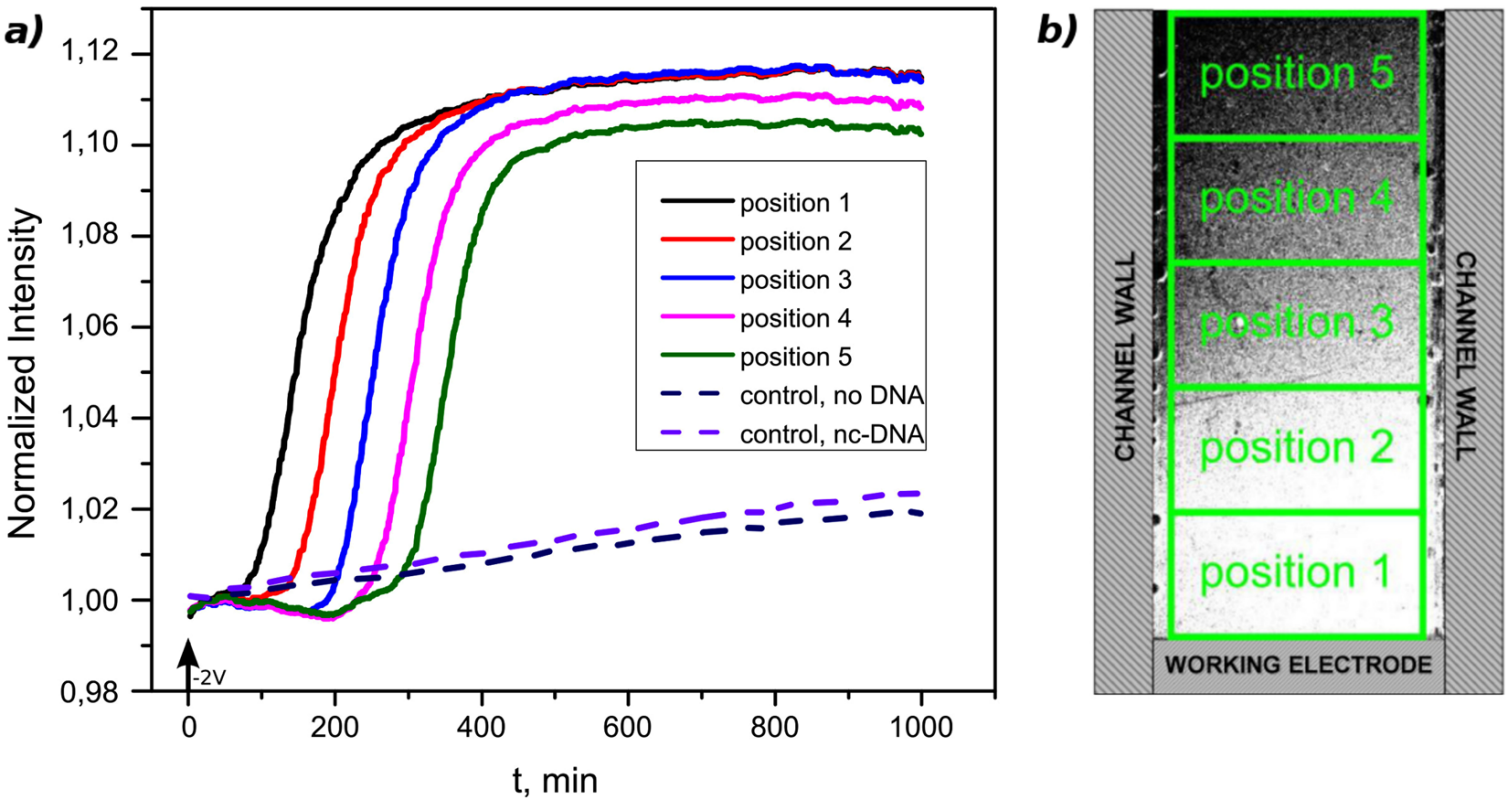
Electrochemical triggering (−2V) of a reaction-diffusion front at t = 0 min. (**a**) The Z-profile taken along the channel length showing propagation of the autocatalytic front as well as two controls with no DNA or non-complementary (nc) DNA on the gold electrode. (**b**) Fluorescent micrograph showing a front propagating in the microfluidic channel and the position at which the Z-profiles are integrated.

We first show how the electrochemical desorption of input makes it possible to trigger the autocatalytic DNA amplification reaction from a specific position in the microfluidic channel and at a specific time. Figure 2 presents the triggering of the molecular system *via* input release at t = 0 min. The electrochemical cleavage of the grafted DNA produces an instantaneous local increase of the trigger strand concentration above the working electrode which is enough to overcome the pseudo-template inhibition effect. As a result, the autocatalytic core starts its exponential production of strands. As expected in such systems, a reaction-diffusion front starts to propagate from the working electrode area and progressively invades the whole channel length. Propagation takes place with uniform velocity, a finding coherent with Zadorin *et al*.^11^, which can be measured at 23 ± 3 µm/min (from Z-profiles, knowing the distance between the different positions). Furthermore, the amplification kinetic are identical for all curves. The system remains in a high state until exhaustion of the dNTPs present in solution.

When an identical experiment is reproduced, but the working electrode is not functionalized with DNA, the fluorescence intensity remains on a base-line level during the complete experiment duration (1000 min - dashed blue curve in Fig. 2). Similar results are obtained in experiments performed with a non-complementary DNA release (dashed purple curve in Fig. 2). We can thus confirm that the electrical pulse of bare gold by itself does not provoke triggering of the reaction (for example by local heating) and only the correct input strand initiates the autocatalysis.

**Figure 3:**
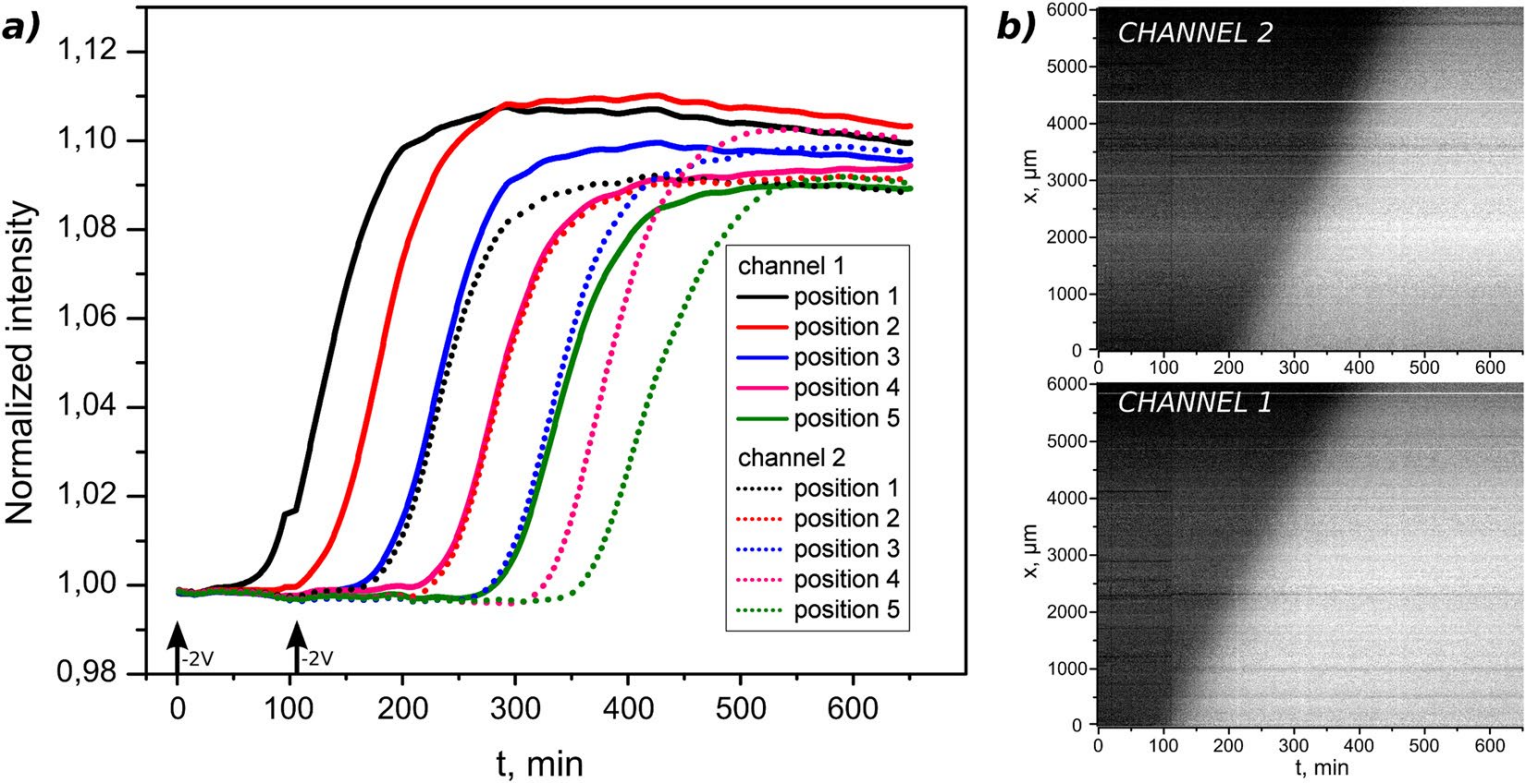
Time-controlled triggering of the molecular system. (**a**) Z-profiles of propagating fronts in the two channels. Channel 1 was triggered at t = 0 min and in channel 2 at t = 108 min as shown by the arrows. (**b**) The kymographs showing independent time controlled triggering in the channels and the constant speed propagation of the fronts.

We wished to illustrate how this principle can be extended to trigger at multiple times or positions in a microfluidic set-up. This ability is of particular importance if one wants to rely on such a strategy to interact with the molecular system in a flexible way. Figure 3 shows kymographs of the reaction-diffusion front propagation in two channels. The first channel is triggered at t = 0 min and the second one at t = 145 min. The fronts propagate at constant velocity in both channels 26 ± 3 µm/min and 28 ± 4 µm/min. Time intervals between amplification curves are consistent with triggering delay which confirms our ability to perform time-controlled triggering.

Even if the system is left without being electrochemically activated, it eventually self-triggers as shown in Fig. S12. The typical delay observed in these cases is well correlated to the thermal stability experiment previously mentioned (Fig. S5). Indeed, as the input DNA strands get released in solution, they reach the minimal triggering concentration established by the drain strands and initiate the autocatalysis. We therefore believe that the only current limitation in the triggering delay comes from the limited stability of the dithiol grafted DNA at 38 °C.

## Conclusion

The use of molecular frameworks to perform information processing tasks is increasingly gaining traction in the community. However, controlling and interacting in real time with these molecular systems in microfluidics is a barrier to study ever more complex scenario and to produce technologies which can be deployed outside the laboratory. In an attempt to solve this problem, we have investigated the electrochemical triggering of PEN-DNA toolbox system using DNA-grafted gold surfaces. This approach was chosen for its potential to be easily transferred to laboratories without microfabrication expertise to maximize its impact. Furthermore, it is not restricted to the PEN toolbox but can also be used with, for example, the strand displacement framework developed by Winfree *et al*.^4^ We demonstrated both time- and space-control of triggering with the investigated system remaining stable during at least 300 minutes under permanent heating at 38 °C. The versatility with which one can produce gold microelectrodes in microfluidic channels or reservoirs makes it possible to use this approach to manage complex spatiotemporal interaction with DNA-based molecular systems. For example, it is possible to rely on multiple sets of electrodes to release different input strands at different locations or times. This could be useful to control the behaviour of the molecular program and coupled with artificial morphogenesis^13^ could enable the dynamical bottom-up assembly of novel materials (*e.g*. DNA-nanoparticle crystals). Our electrochemical actuators could also be used to reprogram molecular systems running in microfluidics by releasing templates instead of input strands. This generic tool is therefore ideally suited to significantly expand the realm of possibilities in the field of DNA-based molecular programming.

## Methods

### Fabrication of working device

**Figure 4:**
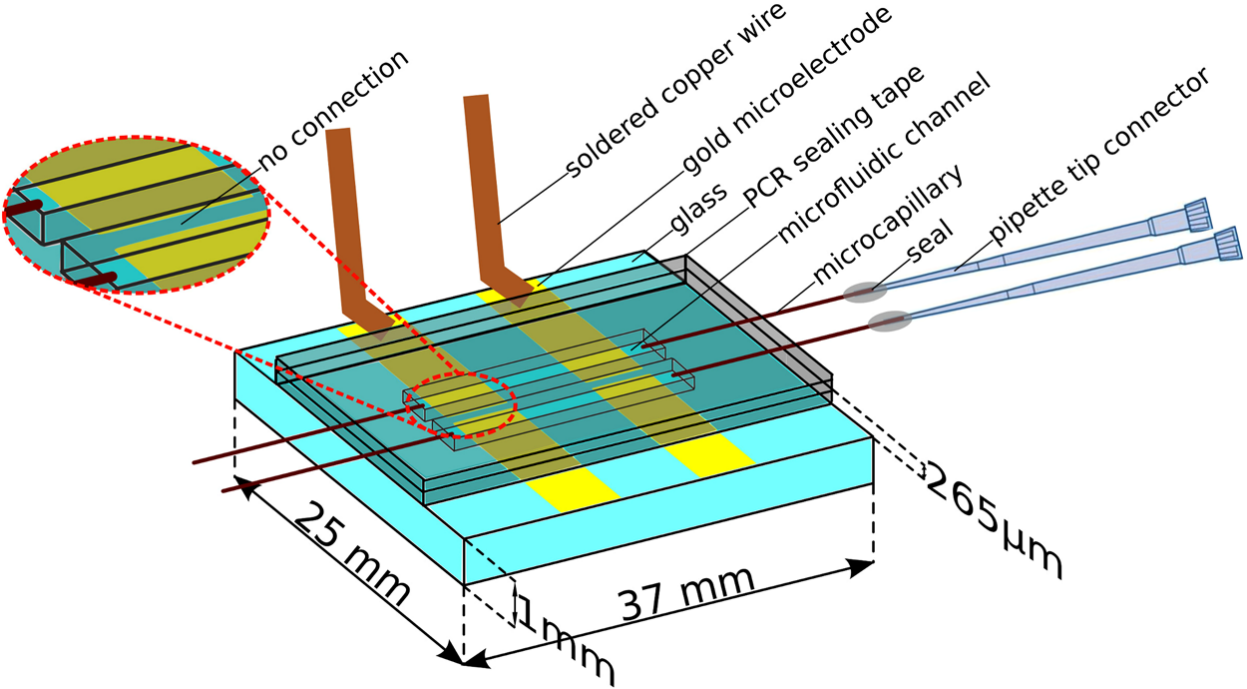
A schematic view of the device showing the two microfluidics channels, each of which is equipped with a working electrode and a counter/reference electrode. The figure also represents the injection set-up used. Note that for clarity only one set of electrode is connected with copper wires.

As depicted in Fig. 4, our device consists of two microfluidic channels perpendicularly aligned with electrodes. The microfluidic part is built from 2 layers of PCR sealing film (Microseal ‘B’, Bio-Rad), assembled on top of a glass substrate. This material was chosen for ease of fabrication and in order to prevent evaporation of the reaction mixture. Electrodes are produced by evaporating 20 nm of titanium followed by 200 nm of gold through a home-made nickel shadow mask onto the glass substrate. The gold electrodes surface was cleaned using a piranha solution (H_2_SO_4_ (98%):H_2_O_2_ (30%) = 1:1) for 20 min (*note that piranha solution must be handled with caution using the appropriate protection equipment*) prior to functionalisation with DNA. Straight channels are manually cut into the adhesive PCR sealing film and perpendicularly aligned to the electrodes. 2 cm length fused silica capillaries (150 μm outer diameter and 75 μm inner diameter, Molex Polymicro Technologies) are inserted on both sides of the channels. The final sealing is obtained by firmly pressing a second PCR film on top of the assembly to ensure removal of all the residual air between the layers. To enable the loading of the reaction mix, 10 µl pipette tips are glued to the glass capillaries on one side. Finally, copper wires are soldered at the end of each electrode to enable their connection to the potentiostat.

### DNA grafting on electrodes

A solution containing 100 nM of DNA-dithiol and 10 µM tris (2-carboxyethyl) phosphine (TCEP – Sigma-Aldrich) in Tris-EDTA (TE) buffer solution (Sigma-Aldrich) was first prepared and kept at room temperature for 2 hours. Subsequently, an equal volume of 2 M NaCl in TE buffer was added to increase the ionic strength of the solution and minimize DNA-DNA strand repulsion. The DNA-dithiol solution was deposited on the cleaned electrode and left to react overnight. The surfaces were rinsed successively with NaCl 1 M, TE buffer and DI water to ensure removal of the excess DNA strands.

### Assembly of the DNA CRN

The reaction buffer is composed of 50 mM NaCl, 10 mM (NH_4_)_2_SO_4_, 10 mM KCl, 8.4 mM MgSO_4_, 0.8 mM of each dNTP [New England Biolabs (NEB)], 0.1% Synperonic F108 (Sigma-Aldrich), 500 μg/mL BSA (NEB), 2 μM Netropsin (Sigma-Aldrich).

DNA oligonucleotides were acquired with HPLC purification (IDT). Their sequences were the following: input, 5′-dithiol CATTCAGGATCG-3′, template, 5′-C*G*A*TCCTGAATG-CGATCCTGAA-3′; pseudo-template, 5′-T*T*T*TTCGATCCTGAATG-3′; reporter, 5′-Cy5 *A*T*TCAGAATGCGATCCTGAAT BHQ2-3′, where * indicates phosphorothioate groups. Just before the experiment’s start, enzymatic mixture was added, 10× solution of which contained: Bst 2.0 WarmStart DNA Polymerase (NEB): 8% of (/20) solution in diluent A (NEB); Nb.BsmI nicking enzyme (NEB): 40%; ttRecJ exonuclease (provided by collaborators): 15% of (/140) solution in diluent A; BSA, 20 mg/ml: 25%; diluent A: 12%.

The following DNA concentrations were used: template – 50 nM, pseudo-template – 10 nM, reporter – 50 nM. All the solutions were prepared in TE buffer.

The reaction mixture was injected in both microfluidic channels. The device was placed on a heating plate (Tokai hit) at 38 °C and the fluorescence signal was measured using an inverted fluorescence microscope (Olympus IX71, 1.25× objective), equipped with CoolLED pE-2 (CoolLED) excitation system. Fluorescence images were processed using the ImageJ software^23^.

### Electrochemical desorption from active surface

Initial electrochemical cleavage of the thiol-bound DNA on the gold surface was investigated vs Ag/AgCl electrodes. For ease of fabrication purposes, a study was thereafter conducted to optimize the conditions for cleavage *vs* a gold electrode. While not ideal from the electrochemical point of view, this is made possible by the fact that the reaction buffer is always the same and that we can afford to work a slight over-potential without going out of the electrochemical window of the solvent. The potential value was thus chosen by observing the release of fluorescently-labelled DNA strand from the surface as a function of applied voltage (Fig. S1). In this study, the DNA input strand release is performed in the reaction buffer by applying a voltage of −2V on the working electrode *vs* gold reference electrode during 30 sec in chrono-amperometry mode using an electrochemical workstation (CH instruments 750E).

## Electronic supplementary material

Supplementary Information

## References

[1] Barabási A-L, Oltvai ZN (2004). Network biology: understanding the cell’s functional organization. Nat. Rev. Genet..

[2] Padirac, A., Fujii, T. & Rondelez, Y. Nucleic acids for the rational design of reaction circuits. *Curr Opin Biotechnol Curr. Opin. Biotechnol*. **24** (2012). 10.1016/j.copbio.2012.11.011 23265857

[3] Wong ASY, Huck WTS (2017). Grip on complexity in chemical reaction networks. Beilstein J. Org. Chem..

[4] Srinivas N, Parkin J, Seelig G, Winfree E, Soloveichik D (2017). Enzyme-free nucleic acid dynamical systems. Science.

[5] Church GM, Gao Y, Kosuri S (2012). Next-Generation Digital Information Storage in DNA. Science..

[6] Adleman LM (1994). Molecular computation of solutions to combinatorial problems. Science.

[7] Benenson Y (2001). Programmable and autonomous computing machine made of biomolecules. Nature.

[8] Seelig G, Soloveichik D, Zhang DY, Winfree E (2006). Enzyme-free nucleic acid logic circuits. Science.

[9] Montagne, K., Plasson, R., Sakai, Y., Fujii, T. & Rondelez, Y. Programming an *in vitro* DNA oscillator using a molecular networking strategy. *Mol. Syst. Biol*. **7** (2011). 10.1038/msb.2010.120 PMC3063689 21283142

[10] Padirac A, Fujii T, Rondelez Y (2012). Bottom-up construction of *in vitro* switchable memories. Proc. Natl Acad. Sci. USA.

[11] Zadorin AS, Rondelez Y, Galas J-C, Estevez-Torres A (2015). Synthesis of programmable reaction-diffusion fronts using DNA catalyzers. Phys. Rev. Lett..

[12] Padirac A, Fujii T, Estévez-Torres A, Rondelez Y (2013). Spatial Waves in Synthetic Biochemical Networks. J. Am. Chem. Soc..

[13] Zadorin AS (2017). Synthesis and materialization of a reaction–diffusion French flag pattern. Nat. Chem..

[14] Zambrano A, Zadorin AS, Rondelez Y, Estévez-Torres A, Galas J-C (2015). Pursuit-and-Evasion Reaction-Diffusion Waves in Microreactors with Tailored Geometry. J. Phys. Chem. B.

[15] Kurylo, I. *et al*. Characterization of peptide attachment on silicon nanowires by X-ray photoelectron spectroscopy and mass spectrometry. *Analyst* **142** (2017). 10.1039/c6an02588a 28239690

[16] Liu M (2015). Remote-Controlled DNA Release from Fe3O4@Au Nanoparticles Using an Alternating Electromagnetic Field. J. Biomed. Nanotechnol..

[17] Li F, Zhang H, Dever B, Li X-F, Le XC (2013). Thermal Stability of DNA Functionalized Gold Nanoparticles. Bioconjug. Chem..

[18] Arinaga K (2007). Controlling the surface density of DNA on gold by electrically induced desorption. Biosens. Bioelectron..

[19] Baccouche A, Montagne K, Padirac A, Fujii T, Rondelez Y (2014). Dynamic DNA-toolbox reaction circuits: A walkthrough. METHODS.

[20] Gines G (2017). Microscopic agents programmed by DNA circuits. Nat. Nanotechnol..

[21] Demers LM (2000). A fluorescence-based method for determining the surface coverage and hybridization efficiency of thiol-capped oligonucleotides bound to gold thin films and nanoparticles. Anal. Chem..

[22] Ricci F, Lai RY, Plaxco KW (2007). Linear, redox modified DNA probes as electrochemical DNA sensors. Chem. Commun..

[23] Schneider CA, Rasband WS, Eliceiri KW (2012). NIH Image to ImageJ: 25 years of image analysis. Nat. Methods.

