# Supplementary material for: Spatiotemporal control of DNA-based chemical reaction network via electrochemical activation in microfluidics
Source: Sci Rep. 2018 Apr 23;8:6396. doi: 10.1038/s41598-018-24659-7 (PMC5913268; doi:10.1038/s41598-018-24659-7)
Supplement: Supplementary file 1 — Supplementary Information [file 41598_2018_24659_MOESM1_ESM.docx]

**Supplementary Information:**

**Spatiotemporal control of DNA-based chemical reaction network via electrochemical activation in microfluidics**

Ievgen Kurylo^1^, Guillaume Gines^3^, Yannick Rondelez^3^, Yannick Coffinier^2^, Alexis Vlandas^1^*

1. BioMEMS, Univ. Lille, CNRS, ISEN, UMR 8520 - IEMN, F-59000 Lille, France. 2. NanoBioInterfaces, Univ. Lille, CNRS, ISEN, UMR 8520 - IEMN, F-59000 Lille, France. 3. Laboratoire Gulliver, Ecole Supérieure de Physique et de Chimie Industrielles, PSL Research University, and CNRS, Paris, France

1. *Electrochemical cleavage of the Gold Thiol bond*

The gold-thiol bond has long been known to be cleavable both oxidatively and reductively. In order to find optimal conditions for electrochemical release in our 2 gold electrodes set-up, a sample with its working gold electrode grafted with Cy5-labelled DNA dithiol (5′-dithiol ATGAGTCAGTAA- Cy5-3′) was prepared and a microfluidic channel was assembled on top of it. The microfluidic channel was filled with 300 mM NaCl solution to obtain an ionicity equivalent to the PEN toolbox.

Thereafter, the potential of the working electrode *vs* the counter/reference one was swept from -0.5 to -2 V with a step 0.1 V and a pulse duration of 10 s for each voltage. Time intervals between the pulses were 90 s. Fluorescence images were taken every 30 s and their intensity measured. The resulting data is shown in Figure S1 (a).

At U = 0 V, while fluorescently labeled DNA is attached to the surface, nearly no fluoresce is measured due to quenching by the gold surface. No fluorescence increase was detected for voltage values lower than -0.9 V. Starting from -0.9 V fluorescence begins slowly raise up after each electrical pulse. A stronger increase starts to be seen around -1.2 V – -1.3 V. Afterwards, each pulse continues to release DNA from the surface until complete depletion. Leaving the potential at -2V for a longer period (40 s) confirms that no further release takes place.


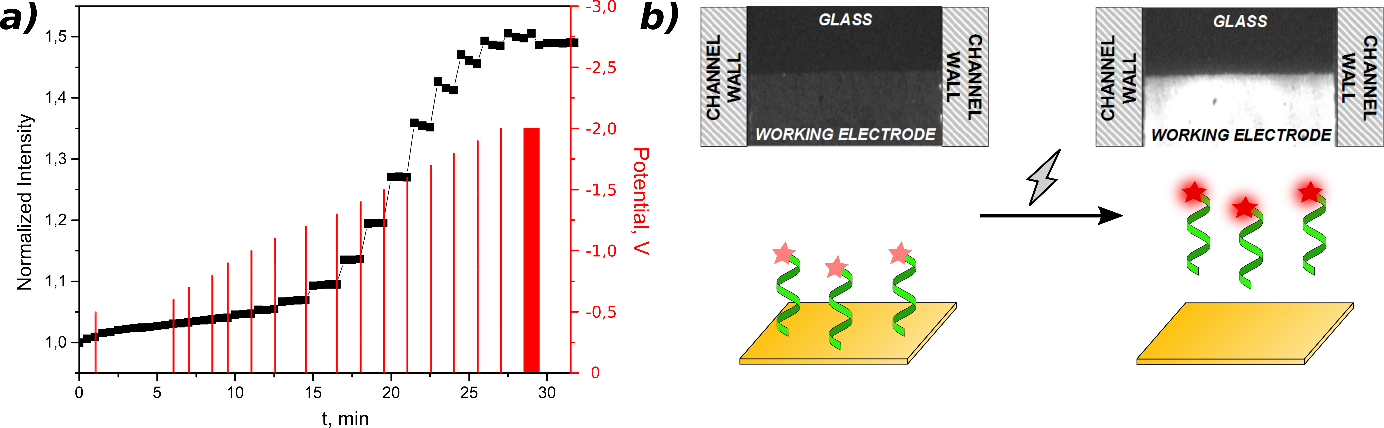


*Figure S1: Electrochemical desorption of fluorescently labelled DNA as a direct observation of the release procedure. (a) Sequential potential increase under continuous fluorescence recording to optimize the release potential. (b) Fluorescence image of sample before and after polarization at -2 V (vs Au) of the working electrode and schematic concept of the mechanism.*

Since -2V *vs* the gold counter electrode is within the solvent window, we choose to apply this potential for 30 s to efficiently cleave the thiol anchor of our DNA from the gold surface and enable them to diffuse away. Figure S1 (b) shows a region of the microfluidic channel where the working electrode occupies the bottom half of the field of view while the top half is glass. One can see the immediate and significant fluorescence increase visible just after the potential was applied.

We also conducted contact angle measurements on gold surfaces before and after DNA immobilization and also after its electrochemical release (Figure S2). We observed substantial decrease of the measured value (from 28±2° to 11±2°) after DNA attachment. These data are in agreement with those previously reported ^1^ and can be explained by the increase of surface hydrophilicity after immobilization. After application of a negative potential (-2 V) to the interface with attached DNA, the contact angle returns back to the initial value (31±2°), which confirms cleavage of Au–S bound under our experimental conditions.

*
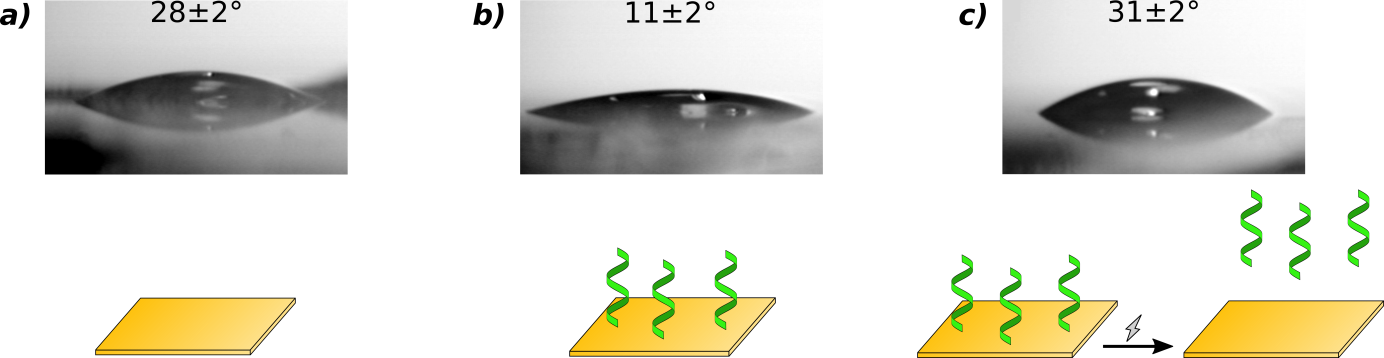
*

*Figure S2: Verification of immobilization and release of DNA from gold surface by measuring the contact angles of bare gold (a), of DNA-functionalized gold surface before (b) and after electrochemical release of attached DNA (c) .*

1. *Surface density determination*

The following procedure (based on Demers *et al.* ^1^) was used to estimate the surface density of grafted DNA on the gold electrodes.

A sample with working gold electrode, grafted with Cy5-labelled DNA dithiol (5′-dithiol ATGAGTCAGTAA- Cy5-3′), was prepared and microfluidic channel was assembled on top of it. The channel was filled with the standard reaction buffer in order to reproduce the usual electrical release conditions (salinity, pH). An electrical pulse of -2 V *vs* the counter electrode was applied during 30 s to the sample and fluorescence intensity, I, as possible close to working electrode was measured as equal to 7540 a.u.

Four identical channels on glass surface were prepared and filled with solutions of the same Cy5-tagged DNA as used for attachment. All solutions were prepared in reaction buffer with DNA at the following concentrations (nM): 1; 5; 10; 15. Fluorescence intensity was measured inside the channels, using the same microscopy settings as for the previous experiment. A calibration curve with equation I = (6937±126) + (328±13)C was obtained (Figure S3). By using this calibration curve, the intensity measured above the gold electrode after release was estimated to correspond to a concentration equal to 1.80±0,04 nM. Using the ImageJ software we measured geometrical parameters working electrode surface area, S, and channel height, h, obtaining 0.11 cm^2^ and 265 µm respectively. The channel volume on top of the working electrode, V, was calculated as V= Sh = 2.9.10^-6^ L. Finally, using the formula Г = CVN_A_/S, where N_A_ is Avogadro’s number, we calculated the surface coverage, Г, as 2.8∙10^10^ molecules/cm^2^.

*
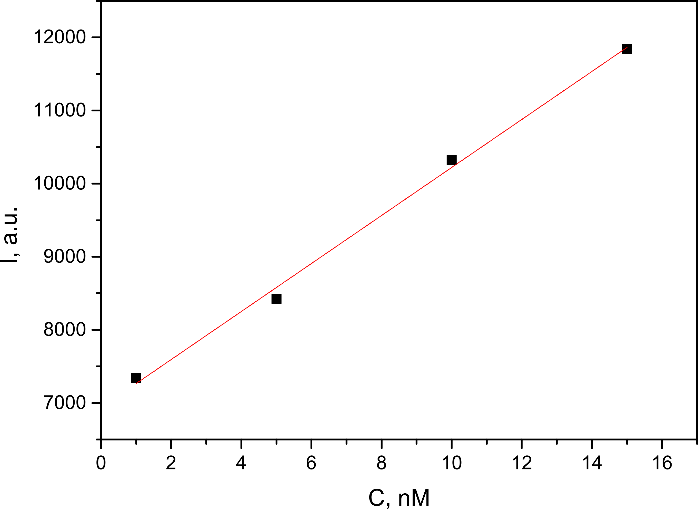
*

*Figure S3: Calibration curve used to determine the grafted DNA surface density (R^2^= 0, 995).*

We also attempted to estimate the maximum amount of DNA which we are able to release from the surface to the reaction medium using bulk experiment (PCR). For this purpose we have conducted the following experiment. Was prepared a gold surface, covered by DNA-input. On top of it was assembled a Parafilm chamber with 10 µl of the reaction buffer. Ag/AgCl reference and carbon counter electrodes were immersed in the chamber. A negative potential of -1,3 V during 30 sec vs Ag/AgCl reference electrode was applied. After that, solution from was removed and mixed with 10 µl of reaction mixture containing enzymes and DNA template. Resulting solution was analysed at 42 °C in PCR thermocycler. In parallel were analysed control solutions, containing the same concentration of template and enzymes and the following concentration of input (nM): 0; 1; 2; 5; 10.


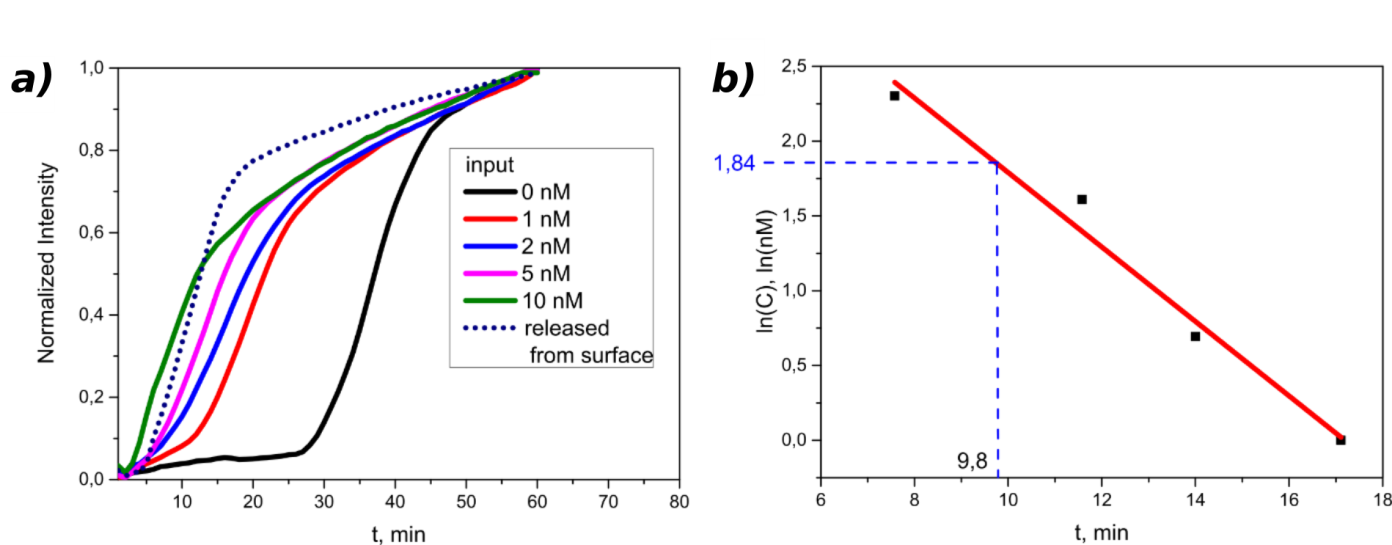


*Figure S4: Determination of the surface coverage by using triggering of autocatalytic reaction in bulk solution w/o surface released input. Raw data (a), processed data for determination of the released input concentration (b). See the text for the details. Each solution contained 50 nM template, 2% of polymerase and 2% of nickase enzyme. The temperature was set to 42 °C.*

From the obtained fluorescence profiles (*Figure S4(a)*), we additionally confirmed our ability to trigger an autocatalysis *via* surface release of input, since the amplification curve, corresponded to the input electrically released (dotted line) is in-between profiles for 5 and 10 nM input and far from the self-start profile (solid black line).

In order to conduct a quantitative estimation of the surface coverage, it was built a calibration curve ln(C)-(t) (*Figure S4(b)*). The value of ***t*** for the input release curve, 9.8 min, was determined from the *Figure S4(a)* and used for the determination of ln(C) value, 1.84, from the *Figure 4(b)*. Therefore, the concentration of input in the analysed sample is equal to e^1.84^ = 6.3 nM. Taking into account the 2 times dilution of the solution after input release (due to the mixing with the equal volume of the reaction mixture), the concentration of DNA, ***C***, which was released in 20 µl of reaction medium volume, ***V***, from the functionalized surface with an area, ***S***, of 0.2 cm^2^, is equal to 12.6 nM. We further calculated the surface coverage, ***Г***, from these data, using the following formula: ***Г =*** $\frac{\boldsymbol{CV}\boldsymbol{N}_{\boldsymbol{A}}}{\boldsymbol{S}}$ . Where $\boldsymbol{N}_{\boldsymbol{A}}$ is Avogadro constant (6.02∙10^23^ mol^-1^). We obtained a surface coverage ~ *10^12^ molecules/cm^2^* which is in good agreement with previously reported surface coverages of DNA-thiol SAM on gold, ranging from 10^10^ to 10^14^ molecules/cm^2^ .

1. *Assembly and optimization of the autocatalytic Chemical Reaction Network (CRN) in bulk*

Prior to launching experiments in microfluidics, it is important to assemble and optimize the conditions of the CRN in bulk.

In particular, one has to adjust the concentration of the pseudo-template ~~concentration~~ to prevent un-triggered start of the autocatalysis while studying the impact on the system kinetics. Two series of solutions with (5 nM) and without (0 nM) input-DNA in the initial reaction buffer were analyzed (experimental details can be found at the end of the Sup. Mat. file). In both series a gradient of pseudo-template concentration – from 5 to 50 nM - was created. Concentrations of all other components (template, enzymes, etc) were equal in every samples with autocatalytic template C = 50 nM, Polymerase 1%, Exonuclease 1% and Nickase 2%. Results of these experiments are shown in Figure S5.


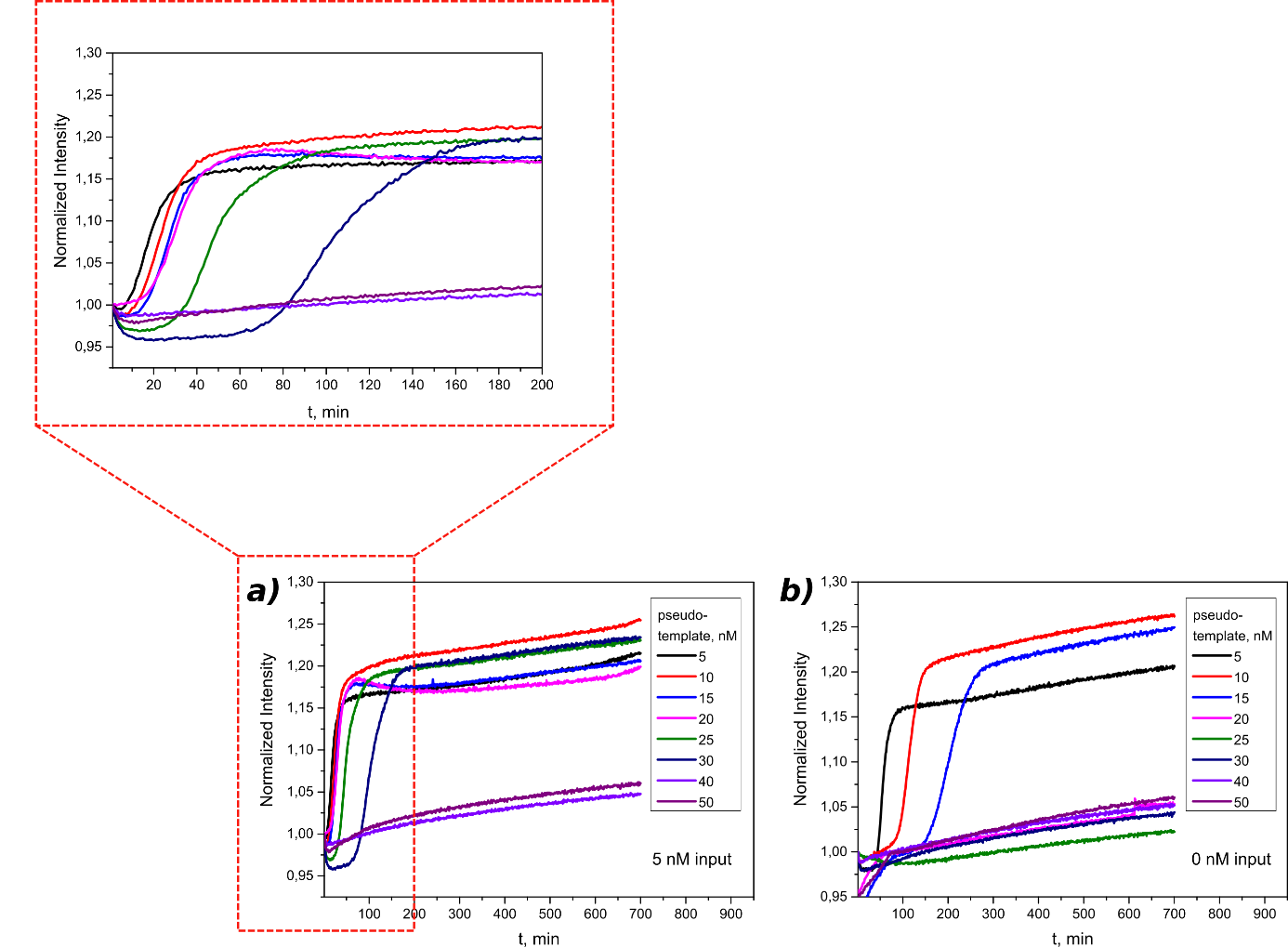


*Figure S5: Fluorescent monitoring of an autocatalytic CRN with various concentrations of pseudo-template at 42°C. a) with 5nM initial input DNA to check in which condition the autocatalysis can be triggered. Additionally, scale from 1 to 200 min is shown. b) without input to check the pseudo template perform its function to prevent self-start.*

The data indicates, that increasing the concentration of pseudo-template leads to delaying the autocatalysis start (exponential intensity growth region) until the eventual complete suppression of the autocatalysis in both experiment’s scenarios – with input in initial reaction mix (fig.S5 a) and without it (fig.S5 b). However, in the first case the pseudo-template has a substantial impact only from relatively high concentrations (higher than 20-25 nM under this particular experimental conditions). At 30 nM for example, the amplification kinetic starts to slow down, while above this value the system never triggers. In contrast, with no initial input, this effect is stronger: for concentrations of pseudo-template higher than 15 nM, no autocatalysis takes place. In other words, at some concentrations range (15-25 nM in this experiment) the autocatalytic CRN is immune to the self-start issues, while at the same time it can still be triggered with input DNA with negligible impacts on its kinetics.

An additional constraint of our approach is to minimize as much as possible the impact of un-designed input thermal-desorption as discussed in the main text. For this purpose CRN parameters – enzymes and pseudo-template concentrations and working temperature – were tuned in a way to make system resistant (or at least less sensitive) to a slow input release. To do so one should place the system as close as possible to the point where the system cannot be triggered with the expected DNA release concentration. Figure S6 shows how pseudo template concentration and working temperature influence this optimum.


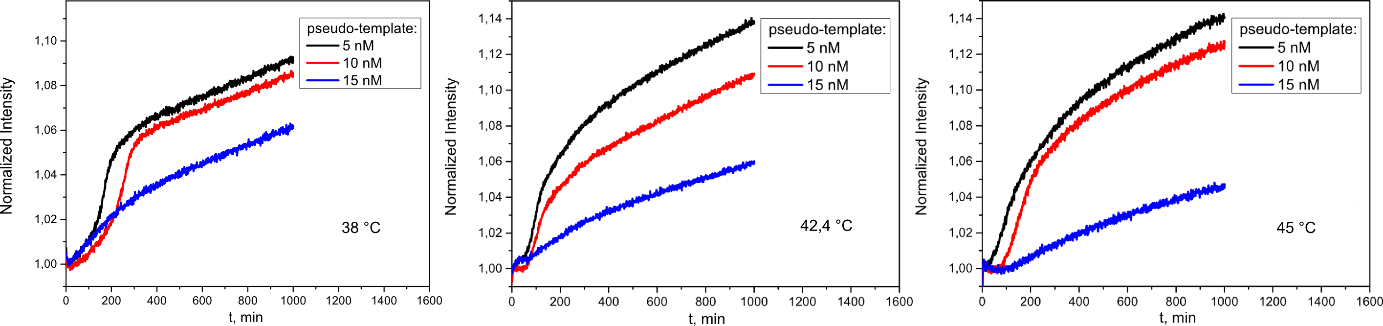


*Figure S6: Finding optimal temperature and pseudo-template concentration for maximal suppression of autocatalysis initiation due parasitic processes (self-start, thermal-desorption).*

Based on these data, temperature of 38 °C and 10 nM pseudo-template concentration were chosen as optimal conditions as under them triggered autocatalysis is strongly delayed (with compared to lower pseudo template concentration), but still taken place. Further increasing of pseudo-template concentration under 38 °C leads to the complete inhibition of the autocatalysis.


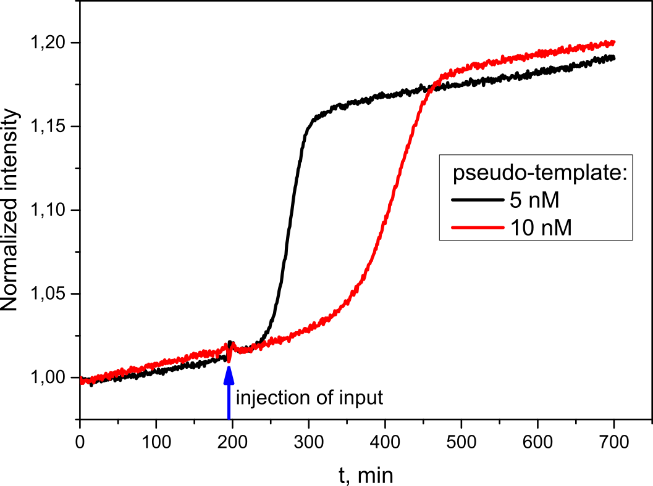


*Figure S7: Activation of CRN at a chosen time in bulk for two pseudo-template concentrations. The system remains stable in its non-catalytic state before injection of input DNA.*

After having successfully assembled the CRN and optimized its parameters, an experiment mimicking the triggering of the autocatalysis as would happen in electrically triggered microfluidics was tested in the bulk. To do so, a non-triggered autocatalytic CRN (50nM template) was held at 38 °C and with 2 pseudo-template concentrations (5 nM and 10 nM) for 194 minutes after which 1 µl of 100 nM input solution (~5 nM final concentration in the reaction mix) was injected. Figure S7 shows how the system does not start before the input injection and how it react to the injection: it takes ~50 min and ~200 min for the system with 5 nM and 10 nM pseudo-template respectively to move into its exponential growth region. This data confirms the CRN stability for >3h time in absence of triggering while being a proof of concept of the delayed activation at a chosen time *via* input-DNA.

1. *CRN in microfluidics without electrochemical triggering*

A first attempt of a spatially triggering the CRN was performed by drying, at localized position within channels, a 0,5 µl droplet of 100 nM input DNA solution.


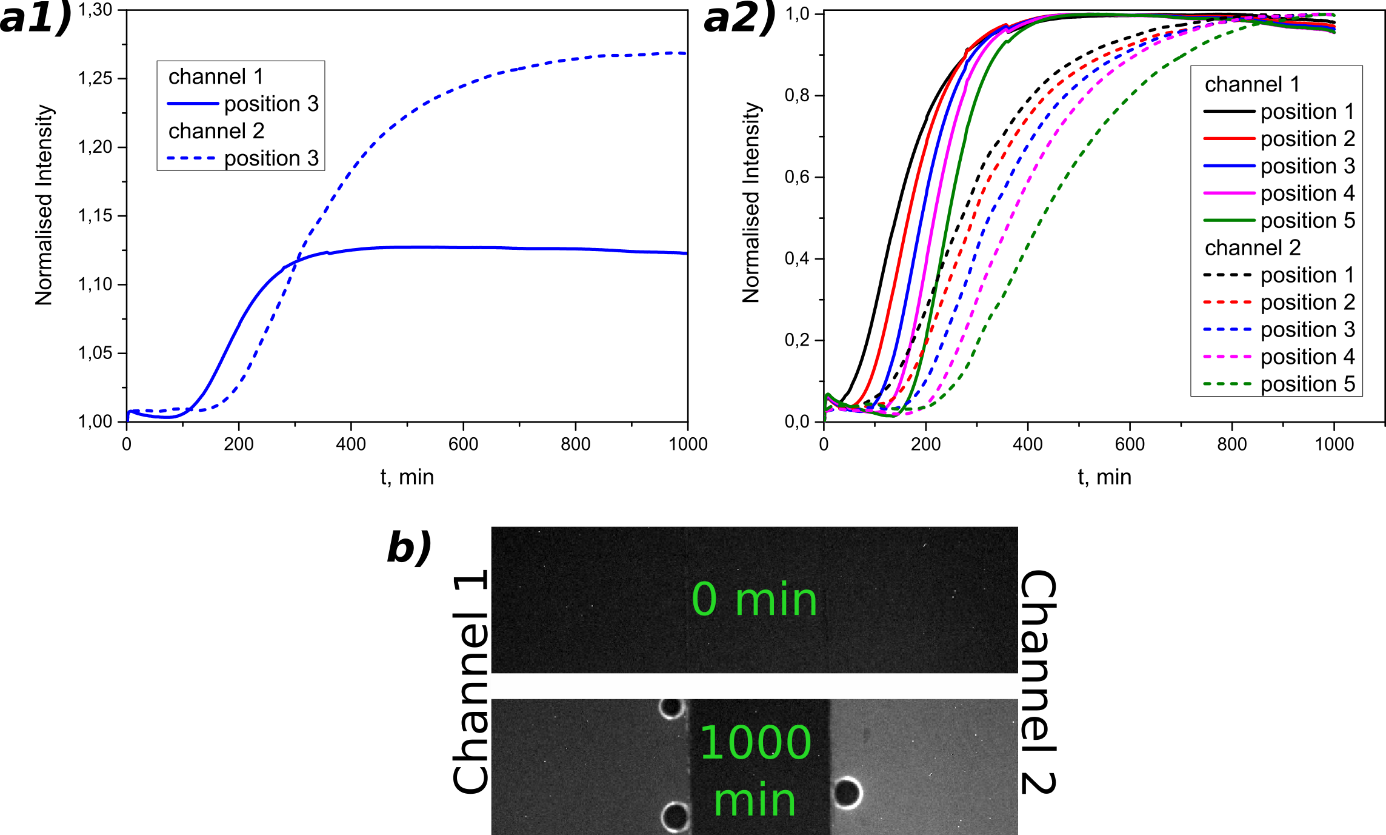


*Figure S8: Verification of impact of reporting reaction on the CRN kinetic. Concentrations of the reporter DNA were following: 50 nM (channel 1) and 100 nM (channel 2). Data were normalized from 0 to 1 (****a2****) for clarity, since original signal increase in channel 2 (****a1*** *and* ***b****) is ~2 times higher.*

Results of this experiment are shown in Figure S8 show clearly reaction-diffusion fronts propagating from the position closest to the dried input region in both channels (50 nM and 100 nM reporter). Increasing the reporter concentration, as expected, leads to higher fluorescence signal (Figures S8 a1 and b). On the same time, however, higher reporter concentration appears to slow down the autocatalytic reaction kinetics (Figure S8 a2). This parameter should be consequently adjusted in a manner which provides sensitive enough reaction monitoring and, at the same time, does not change the reaction kinetics too severely. We will demonstrate farther that the reporter concentration 50 nM under this experimental conditions is satisfactory in terms of CRN behavior and detection sensitivity.

Further experiments were performed in order to check that the input DNA release cannot, by hybridizing and diffusion, produce changes in the reporter strand fluorescence intensity which resemble the front propagation observed. As previously, a 0,5 µl droplet of 100 nM input solution was locally deposited in two separate microfluidic channels. The first channel was filled with the reaction mixture containing all the components of CRN and the second one with the same solution, but without the autocatalytic template. Time evolution of intensity along the channels was recorded and is shown in Figure S9.


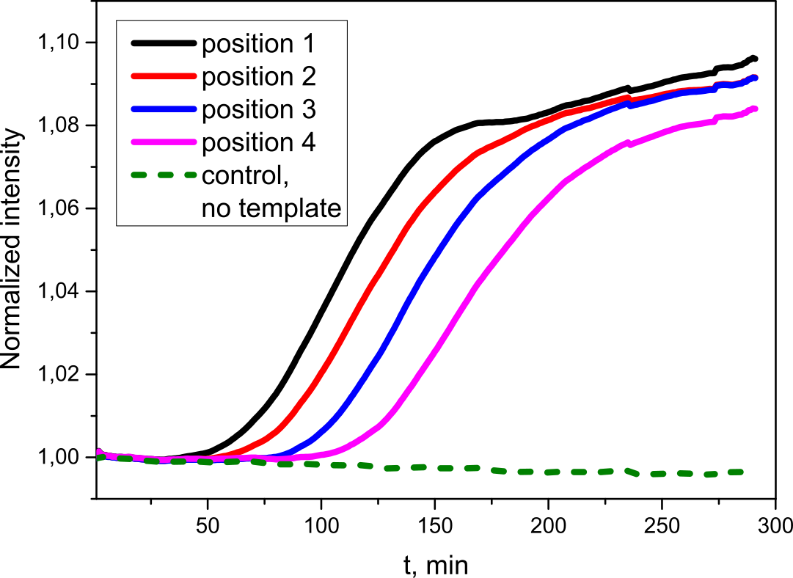


*Figure S9: Control for initial input DNA release impact on reporter strand fluorescence. Profiles in two channels respectively with (position 1-4) and without the autocatalytic template. Reporter strand concentration is 50 nM.*

1. *Impact of the surface composition on CRN behavior*

Since our electrochemical triggering requires presence of gold electrodes in microfluidic channels, we investigated whether or not this material has an impact on reaction kinetics. For this purpose we launched an auto-catalysis (initial input concentration 5 nM) in channel assembled over a half glass/half bare gold surface. The experiment was performed for two concentrations of reporter strand.


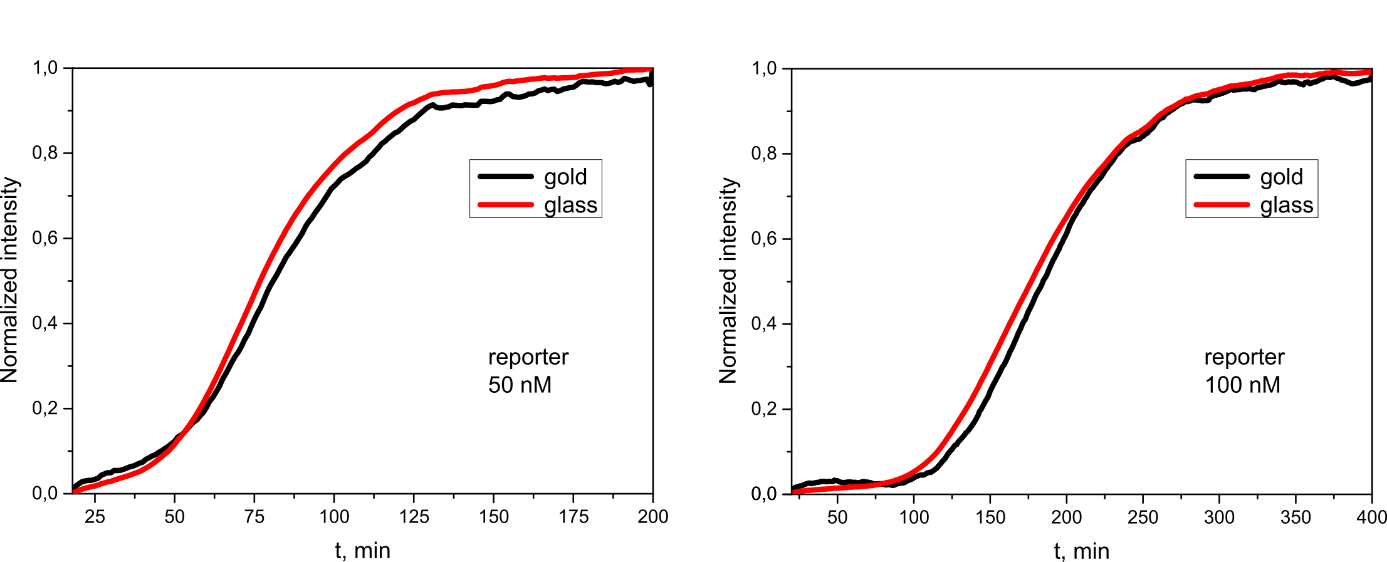


*Figure S10: Impact of a gold surface on autocatalytic and reporting reactions. Note that for these particular data was applied 0-1 normalization, since due to the reflective properties of the gold surface original signal on it is substantially higher rather than on transparent glass.*

Figure S10 indicates fairly identical reaction kinetics on gold and on glass surface for both reporter concentrations. We can consequently conclude, that integration of gold electrodes in our microfluidic setup does not have a detrimental effect on the CRN or our ability to monitor it.

1. *Undesigned system self-triggering*

To assess the thermal stability of our thiol grafted DNA, a monitoring experiment was conducted. As previously, the gold electrode was functionalized with Cy5-tagged ssDNA. The microfluidic channel was filled with the reaction buffer and was placed on the heating plate at 38 °C to reproduce the actual experimental conditions. Fluorescence intensity on top of the electrode was measured during 1000 minutes at 1 minute intervals. Results of experiment are shown on Figure S11 and demonstrate the layer stability for at least 240 min.


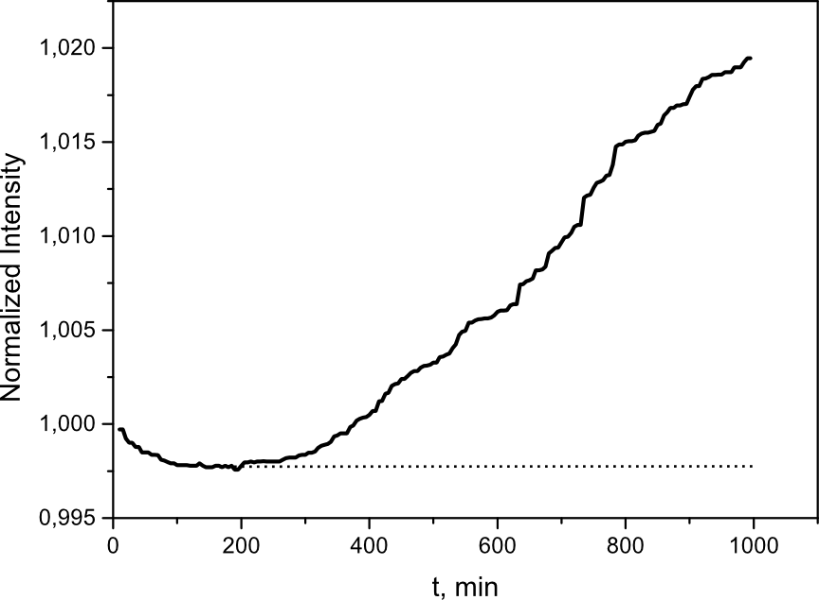


*Figure 11: Fluorescence monitoring of the thiol grafted DNA at 38°C. After 240 min without increase, the fluorescence starts to rise.*

The experiment described in main text was also reproduced for 2 identical channels, one of which was electrically triggered at t = 0 min, and the second one left under the same conditions, but without electrical triggering. For the first channel, a similar behavior seen in Figure 3, was observed, while fluorescence profile in the second channel remains stable during 5 hours (300 min). After that period, the fluorescence signal starts to increase from the working electrode area, which is probably due to a triggering of the system due to thermal desorption of attached input (Figure S12).


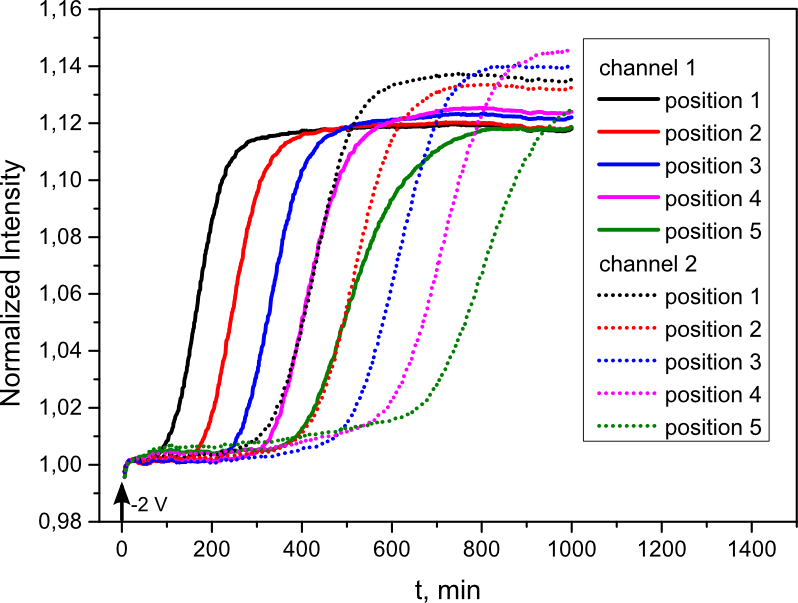


*Figure S12: An example of the involuntary self-triggering of the autocatalytic system (channel 2). The system in Channel 1 was triggered electrochemically at t = 0.*

1. *Experimental methods*

The reaction buffer was prepared by mixing 50 mM NaCl, 10 mM (NH4)2SO4, 10 mM KCl, 8,4 mM MgSO4, 0,8 mM of each dNTP [New England Biolabs (NEB)], 0.1% Synperonic F108 (Sigma-Aldrich), 500 μg/mL BSA (NEB), 2μM Netropsin (Sigma-Aldrich).

DNA oligonucleotides were acquired with HPLC purification (IDT). Their sequences were the following: input, 5’-dithiol CATTCAGGATCG-3’, template, 5′-C*G*A*TCCTGAATG-CGATCCTGAA-3′; pseudo-template, 5′-T*T*T*TTCGATCCTGAATG-3′; reporter, 5′- Cy5 *A*T*TCAGAATGCGATCCTGAAT BHQ2-3′, where * indicates phosphorothioate groups. Just before the experiment’s start, enzymatic mixture was added, 10 x solution of which contained: Bst 2.0 WarmStart DNA Polymerase (NEB): 8% of (/20) solution in diluent A (NEB); Nb.BsmI nickase (NEB): 40%; ttRecJ exonuclease (provided by collaborators): 15% of (/140) solution in diluent A; BSA, 20 mg/ml: 25%; diluent A: 12%.

1. *Data Availability*

The datasets acquired or generated during the current study are available from the corresponding author.

REFERENCES

1. Ladik, A. V, Geiger, F. M. & Walter, S. R. Immobilization of DNA onto Gold and Dehybridization of Surface-Bound DNA on Glass. *Undergraduate Researcher Graduate Student Mentor.* **7,** (2010).

2. Demers, L. M. *et al.* A fluorescence-based method for determining the surface coverage and hybridization efficiency of thiol-capped oligonucleotides bound to gold thin films and nanoparticles. *Anal. Chem.* **72,** 5535–41 (2000).
